# The shoaling behavior of two cyprinid species in conspecific and heterospecific groups

**DOI:** 10.7717/peerj.3397

**Published:** 2017-05-30

**Authors:** Zhong-Hua Tang, Hui Wu, Qing Huang, Lu Kuang, Shi-Jian Fu

**Affiliations:** Laboratory of Evolutionary Physiology and Behavior, Chongqing Key Laboratory of Animal Biology, Chongqing Normal University, Chongqing, China

**Keywords:** Heterospecific shoal, School structure, Collective behavior, School, Cyprinidae

## Abstract

Mixed-species shoals of fish are frequently found in the field; however, little is known about individual-level interactions within these groups. We examined the collective motion of two cyprinid species (Chinese bream, *Parabramis pekinensis*, and qingbo, *Spinibarbus sinensis*) that occupy partially overlapping habitats but differ in social behavior (high vs low aggressiveness) and preferred flow regime (slow vs fast water velocity). We extracted measures of collective motion from video recordings of eight replicate groups of four individuals of either Chinese bream or qingbo (conspecific group) or two Chinese bream plus two qingbo (heterospecific group). Chinese bream in conspecific groups showed lower percent time moving and mean swimming speed but a similar speed while moving as compared to the qingbo conspecific groups. However, the difference in mean swimming speed and percent time moving vanished in the heterospecific group as Chinese bream elevated their swimming activity to coordinate with qingbo. This finding suggests that the two species may share similar interaction rules regarding shoaling behavior. The conspecific groups of qingbo exhibited a greater distance between group members than Chinese bream, suggesting a difference in cohesion. However, the inter-individual distances of all fish were similar in the heterospecific group. Qingbo in the heterospecific group swam more frequently at the front compared to Chinese bream, possibly due to their higher activity level. We also measured the startle response to an artificial stimulus and found that there was no significant difference among groups. In conclusion, the present study demonstrated that in the heterospecific groups, Chinese bream elevated their percent time moving while qingbo decreased their inter-individual distance to achieve consistent collective movement; thus, the two species showed similar behavior in the mixed-species group.

## Introduction

Group living is commonly observed among fish species in nature, and approximately half of all known fish species school for part or all of their lives (Shaw, 1978). Fish derive many benefits from group living, including defense against predators and enhanced foraging success (Krause & Ruxton, 2002). The behavior of individuals and the interaction between them produce group-level behavior and determine how fish move, transfer information and make decisions between individuals, shaping the properties of fish schools (Herbert-Read et al., 2011; Herbert-Read et al., 2013; Shelton et al., 2015; Suriyampola et al., 2016). The most frequently studied properties of fish groups include cohesion, which is usually indicated by neighbor distance, and coordination, as indicated by the synchronization of swimming activity and polarity of orientation (Delcourt & Poncin, 2012; Miller & Gerlai, 2012; Herbert-Read et al., 2013). In addition to such properties, the positions that individuals take relative to others in a group have important evolutionary and ecological consequences because spatial positions may have both benefits, costs (Krause & Ruxton, 2002). The morphological, physiological and behavioral differences among individuals may all influence the position adopted by an individual within a group; therefore, individuals within groups may be expected to modify their positions relative to neighbors as a function of their internal state (Killen et al., 2012). The ecological benefits, costs of group living and the spatial distribution pattern vary with species due to differences in predation stress, food availability, and ecological habits (Gimeno et al., 2016; Suriyampola et al., 2016). Thus, school properties, such as cohesion, coordination, and spatial distribution pattern might vary by fish species.

Individuals within groups benefit when they are phenotypically and behaviorally similar (Farine, Montiglio & Spiegel, 2015). Many fish species prefer conspecific shoals to heterospecific shoals based on both field observations and laboratory measurements (Krause et al., 2000a; Ward & Hart, 2003; Blakeslee et al., 2009). However, mixed-species shoals are also frequently found among a wide range of animal taxa and provide advantages in terms of enhanced foraging efficiency and predator avoidance (Goodale et al., 2010; Rodgers, Downing & Morrell, 2015; Kleinhappel et al., 2016). Although it has long been suggested that the presence of multiple species might change individuals’ behavior and position within the shoal (Allan, 1986), the shoaling behaviors of mixed-species groups have received little attention (Morse, 1977; Ward, Axford & Krause, 2002; Quinn et al., 2012). Thus, little is known about individual-level interactions within heterospecific groups and the mechanisms mediating their structure (Kleinhappel et al., 2016). Acquiring information from other species of the same trophic level has been suggested to be widespread among animals and a possible driving factor in the formation or maintenance of temporary or stable heterospecific groups (Goodale et al., 2010). Interspecies differences in information transfer, along with the relatively profound differences in morphology, physiology, and behavior compared to the differences within species, may alter shoal properties, such as structure and distribution pattern, producing new ecological benefits and costs within a heterospecific group (Krause et al., 2000b; Goodale et al., 2010). Furthermore, animals show greater boldness and hence alter their response to predator attack when in the presence of conspecifics (Magurran, 1986; Dill & Ydenberg, 1987). However, the presence of heterospecifics may have a different effect on the response to predation threat, as the efficient transmission of information regarding a predator threat by neighbor copying might relate to the group species composition and predation risk; hence, the cost may vary with species due to the differences in anti-predation strategies (Goodale et al., 2010).

In the present study, we tested whether school properties such as cohesion, coordination and spatial structure (suggested by the percentage of time a given member swims at the front of the group) as well as their response to fright stimuli vary between fish species, and if so, how individuals of different species within a heterospecific group adjust their behavior, thereby altering the shoal’s properties compared to a conspecific shoal. To fulfill our goals, we selected Chinese bream (*Parabramis pekinensis*) and qingbo (*Spinibarbus sinensis*), two cyprinid fish species, as experimental models. They are both herbivorous fish species that prefer shoaling (Fu, 2016; Killen et al., 2016). However, some key differences exist. Chinese bream, although frequently found in fast-flowing water, prefer slow water, whereas qingbo prefer to live in fast currents (Yan et al., 2013; Fu et al., 2014). Furthermore, Chinese bream are 10 times more aggressive than qingbo according to chasing and nip data from our pilot experiment. Based in part on the methods of a previous study (Delcourt & Poncin, 2012), we measured the cohesion (as suggested by inter-individual distance), synchronization of swimming speed, polarization, and fright responses of groups composed of four Chinese bream (conspecific group), four qingbo (conspecific group) or two Chinese bream individuals plus two qingbo individuals (heterospecific group). A previous study found that cyprinids, such as the Chinese bream and qingbo in the present study, show typical shoaling behaviors in groups as small as four individuals (Fu et al., 2016). We anticipated that (1) the swimming activity, cohesion and coordination of qingbo would be higher while the percent of time swimming at front lower than that of the Chinese bream due to their difference in preferred habitat and social behavior; (2) the cohesion and coordination and startle response to stimuli of the heterospecific group would be lower than those of conspecific group due to the decreased information flow efficiency between groups members.

## Materials and Methods

### Ethics statement

This study was performed in strict accordance with the recommendations in the Guide for the Care and Use of Laboratory Animals of the Key Laboratory of Animal Biology of Chongqing (Permit Number: Zhao-20140622-01), China.

### Experimental fish and holding conditions

Juvenile Chinese bream (body mass: mean ± SD = 4.70 ± 1.89 g, standard body length: 6.39 ± 0.80 cm, *N* = 48) and qingbo (body mass: 4.91 20 ± 1.23 g, standard body length: 6.14 ± 0.51 cm, *N* = 48) with similar body sizes were obtained from local fishermen. The two fish species were maintained in separate recirculating water tank systems (length × width × height: 1.5 × 0.6 × 0.5 m) that included a biological filter and an ultraviolet lamp at Chongqing Normal University for 2 wk prior to experimentation. During this time, the temperature of the dechlorinated freshwater was maintained at 25.0 ± 0.5 °C (which is the common temperature of the qingbo’s habitat in summer), and oxygen saturation was maintained above 90%. The photoperiod was 12 L:12 D. One-tenth of the water was replaced daily to maintain good water quality. Throughout the experimental period, the fish were fed daily to satiation with commercial food until 48 h before the experimental trials.

### Experimental protocol

A round experimental arena (diameter 100 cm; depth 25 cm) constructed of 1-cm-thick acrylic was filled to a depth of 7 cm with dechlorinated water. The arena was lit by fluorescent lights and visually isolated by an opaque plastic curtain to prevent external disturbances to the fish. A webcam (Logitech Pro 9000; Logitech Company, Suzhou, China) placed directly over the arena video recorded the movements of the fish groups (at 30 frames per second). The school consisted of four Chinese bream individuals (conspecific group), four qingbo individuals (conspecific group) or two Chinese bream plus two qingbo individuals (heterospecific group). The experiment was performed from 9:00 to 17:00, and the video recording process lasted three days (one treatment group per day). During the experiment, the experimental fish were gently netted from the holding tank into a plastic beaker without exposure to air, transferred to the experimental arena, and carefully released into the center of the arena. The fish were acclimated for 5 min and then video recorded for 10 min. We found that the shoaling traits of the two species stabilized after 5 min in the experimental arena, similar to zebrafish shoals (Miller & Gerlai, 2012). The fish were then stimulated by tapping with fingers the same location of the inner wall of the experimental arena three times with identical force by the same experimenter while video recording. The interval between stimuli was 20 s. This type of stimulus was selected because tapping produced a short-lived stress response in the fish, and such stimuli are an appropriate way to discover behavioral differences (Wilson et al., 1993). After each experimental run, the experimental tank was carefully cleaned and refilled with fresh dechlorinated water and the fish were removed and not reused for subsequent measurements. Eight repetitions of each treatment were video recorded, and the locomotive trajectories were then analyzed.

### Measures of collective motion

Videos were converted from .wmv to .avi format using Format Factory (http://format-factory.softonic.cn) and imported into the automated tracking software program idTracker (v 2.1) (see Pérez-Escudero et al., 2014 for a full description of the tracking software). The error rate was lower than 1% of the total trajectories in the present study due to the different morphology between two species. These programs automatically tracked the centroid of each fish in each trial and gave all *x* and *y* coordinates of each fish in each video. The 10-min coordinates (i.e., 6–15 min after 5 min of acclimation) were used for analysis of the dependent variables because we found that shoaling can be characterized in session lengths as short as 10 min (Fu et al., 2016). The *x* and *y* coordinates in pixels were converted to centimeters (Killen et al., 2012). In addition, the raw trajectories were noisy due to the changes in body shape of the fish during swimming and errors of the tracking system. The trajectories were therefore smoothed using a weighted moving average with a window width of 0.5 s, and the new interval between two successive frames was 0.5 s (Miller & Gerlai, 2012). The formulas for the tested scale of the frames, as used in previous studies, are described below (see review in Delcourt & Poncin, 2012), and the mean values are reported.

The swimming activity of the experimental group were evaluated by the mean swimming speed, speed while moving and percent time moving, which was calculated by following formulas:

Instantaneous speed (v, cm s^−1^):

(1) \begin{eqnarray*}v(t)=\sqrt{(x(t)-x(t-1))^{2}(y(t)-y(t-1))^{2}}/d\end{eqnarray*}

where *x*(*t*), *x*(*t* − 1) and *y*(*t*), *y*(*t* − 1) are the *x* and *y* coordinates of the measured fish at time *t* and the previous frame (*t* − 1) and *d* is the length of the time interval (i.e., 0.5 s). The fish were considered moving when the instantaneous swimming speed was higher than 1.75 cm s^−1^, and resting when the speed was lower.

Mean swimming speed: the average instantaneous speed of each individual over the whole measurement period (i.e., 10 min).

Speed while moving: the average instantaneous speed of individuals while their instantaneous speeds were higher than 1.75 cm s^−1^.

Percent time moving: the percentage of time when individuals’ instantaneous speeds were higher than 1.75 cm s^−1^.

Synchronization of speed (Sv): this measurement quantifies the correlation between scalar speeds and can be calculated by the following formula (Delcourt & Poncin, 2012):

(2) \begin{eqnarray*}{S}_{v}=1- \left\vert \frac{{v}_{i}-{v}_{j}}{{v}_{i}+{v}_{j}} \right\vert \end{eqnarray*}

where *v*_*i*_ and *v*_*j*_ are the instantaneous swimming speeds of each pair of individuals within the group.

Polarization while moving: this measurement quantifies the degree of alignment of a shoal of fish when swimming and can be defined by this formula (Miller & Gerlai, 2012; Gimeno et al., 2016):

(3) \begin{eqnarray*}p(t)= \frac{1}{N} \left\vert \sum _{i=1}^{N}{v}_{i}(t) \right\vert \end{eqnarray*}

where *v*_*i*_(*t*) is the unit movement vector of fish *i*. The orientation is from the position at time *t* − 1 (previous frame) to that at time *t*. *N* is the number of individuals of a group (i.e., 4). The values of the polarization range from 0 to 1. When all individuals are aligned in the same direction, polarization equals 1; when the unit movement vectors are of the individuals cancel each other out, polarization equals 0.

Inter-individual distance (IID, cm): this measurement quantifies the cohesive tendency of a group using the following formula:

(4) \begin{eqnarray*}II{D}_{i}(t)= \frac{1}{N-1} \left\vert \sum _{i\not = j}{D}_{ij} \right\vert \end{eqnarray*}

where ${D}_{ij}=\sqrt{({x}_{i}-{x}_{j})+({y}_{i}-{y}_{j})^{2}}$ and *x*_*i*_, *y*_*i*_ and *x*_*j*_, *y*_*j*_ are the *x* and *y* coordinates of any individual fish and its neighbors, respectively.

The spatial distribution pattern was evaluated by the inter-individual variation within a shoal (i.e., the coefficient of variation (CV) among group members) according to the percent time swimming at the front (when all members of a shoal were moving). The range of CV was from 0 to 2. When each fish spent exactly 25% of the time at the front, the CV would be 0 and if one fish spent 100% of the time at the front the CV would be 2. A fish was identified as swimming at the front of the shoal by two criteria: firstly, to select the fish that swimming at the front, the distance between the centroid of the group (the mean of the coordinate of four fish) at time *t* − 1 and that of the fish at time *t* must be maximal; secondly, to select the fish moving in approximately the same direction with the group members, the separation angle (SA) between the movement direction of the group (the centroid of the group points from its position at time *t* − 1 to that at time *t*) and that of the fish must be less than 20°. SA is defined as the angular difference between the vector of any one individual (A_1_) within a given group and the vector of the group (A_2_).

(5) \begin{eqnarray*}SA={A}_{1}-{A}_{2}\end{eqnarray*}

where $A=\arctan \frac{y(t)-y(t-1)}{x(t)-x(t-1)} ,$ *x*(*t*) and *y*(*t*) are the coordinates of an individual fish or the group, and *x*(*t* − 1) and *y*(*t* − 1) are the coordinates of previous frames (length of the time interval was 0.5 s).

An individual was considered to have performed a startle response if the instantaneous speed reached at least 20 cm s^−1^ for at least 5 s. This is based on a previous study showing that an instantaneous speed higher than 20 cm s ^−1^ was occurred less than 4% of the time under the undisturbed condition (Wu et al., 2017).

Startle response consistency (C, %)

(6) \begin{eqnarray*}C= \frac{{N}_{b}}{{N}_{s}} \times 100\text{%}\end{eqnarray*}

where *N*_*b*_ is the number of all group members that showed an identical response, i.e., all responded or all failed to respond to the stimulus, and *N*_s_ is the total number of fright stimulus trials.

### Data analysis

SPSS 17.0 (SPSS Inc., and IBM, Chicago, IL, USA) was used for data analysis. *P* values <0.05 were considered statistically significant, and all data are presented as the mean (±S.E.) of the group. One-sample Kolmogorov–Smirnov test results indicated that all data were normally distributed. The differences in all measured variables among the three treatment groups (Chinese bream conspecific group, qingbo conspecific group and heterospecific group) were examined by one-way analysis of variance (ANOVA) using the mean values of the groups (the video conversion of one qingbo conspecific group failed). The differences in the variables in the heterospecific group were determined by a linear mixed model (LMM) with individual values. Duncan’s multiple range test or paired *t*-test comparison was performed after ANOVA or LMM if necessary.

## Results

### Swimming speed and its synchronization

**Figure 1:**
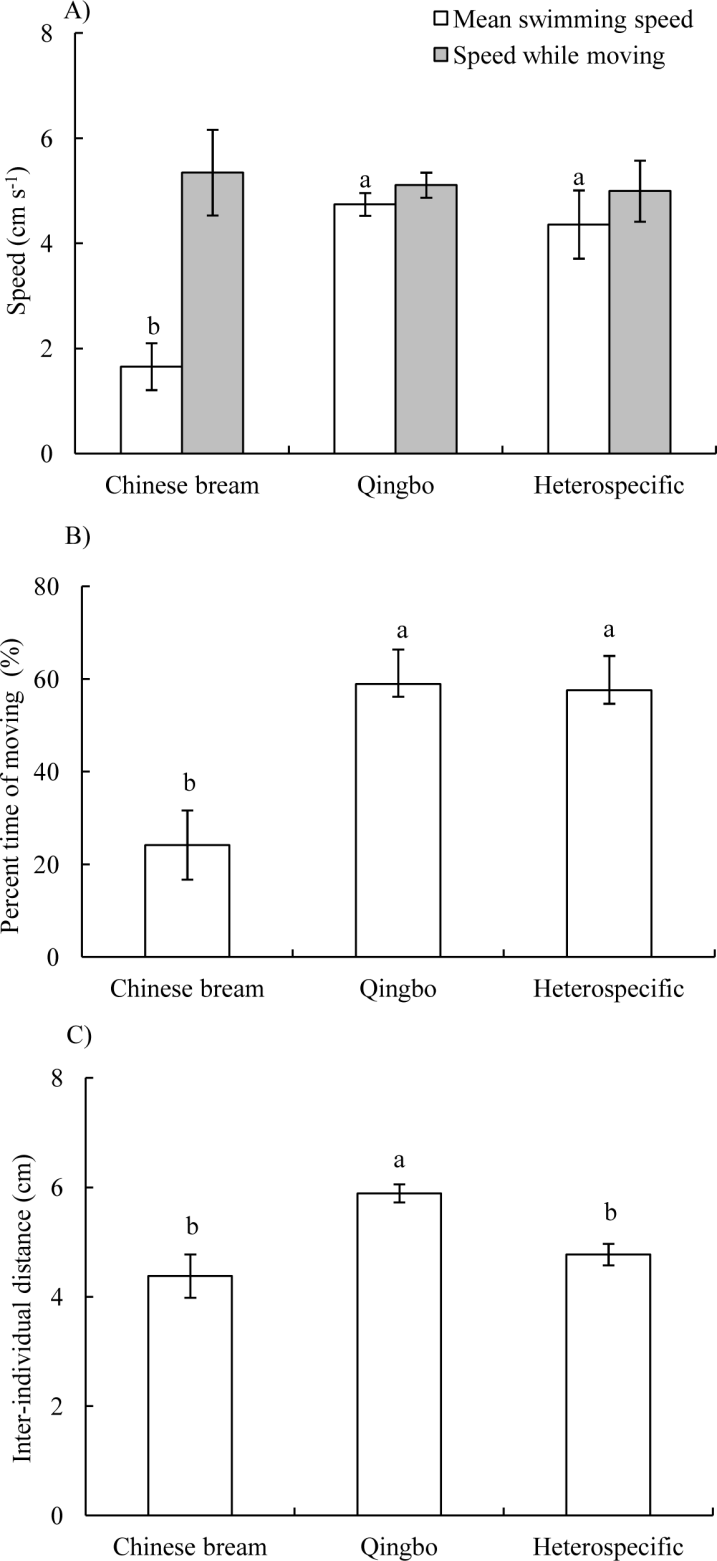
Mean swimming speeds and speed while moving (A), percent time moving (B) and inter-individual distance (C) (Mean ± S.E., *N* = 8 except for qingbo *N* = 7) of the fish in the different treatment groups. a and b indicate a significant difference between the two treatments, whereas the same letter indicates no statistically significant difference by Duncan’s multiple-range test.

In both the conspecific and heterospecific groups, the four fish often formed a group and primarily swam along with the wall of the arena. The mean swimming speed of qingbo in the conspecific group and heterospecific group was significantly higher than that of Chinese bream in the conspecific group (*P* < 0.001), whereas there was no significant difference in speed while moving among the three groups (ANOVA, *F*_2,20_ = 0.088, *P* = 0.916) (Fig. 1A). The percent time moving was significantly lower in the conspecific Chinese bream group than in the conspecific qingbo group and heterospecific group (ANOVA, *F*_2,20_ = 15.504, *P* < 0.001) (Fig. 1B). There was no significant difference in synchronization of speed among the three groups (Table 1).

In the heterospecific group, neither mean swimming speed nor speed while moving differed significantly between qingbo and Chinese bream (Table 2). Furthermore, neither the percent time moving of the Chinese bream and qingbo in the heterospefic group nor synchronization of speed among the Chinese bream, qingbo and Chinese bream-qingbo in the heterospefic group differed significantly (Table 2).

**Table 1:** Statistical analysis of the differences in the measured behavioral variables among the three treatment groups by one-way analysis of variance (ANOVA).

Variable	Chinese bream	Qingbo	Heterospecific	ANOVA
Synchronization of speed	0.82 ± 0.02	0.78 ± 0.05	0.84 ± 0.00	*F*_2,20_ = 0.988*P* = 0.390
Polarization	0.92 ± 0.02	0.89 ± 0.01	0.91 ± 0.01	*F*_2,20_ = 0.949*P* = 0.404
CV of percent time swimming at the front	0.75 ± 0.09	0.52 ± 0.09	0.79 ± 0.09	*F*_2,20_ = 1.614*P* = 0.224
Percent startle response (%)	50.00 ± 18.90	78.57 ± 11.48	87.50 ± 6.68	*F*_2,20_ = 2.174*P* = 0.140
Startle response consistency (%)	100.00 ± 0.00	57.14 ± 18.90	62.50 ± 18.30	*F*_2,20_ = 2.356*P* = 0.121

**Table 2:** Statistical analysis of the differences in the measured variables between individuals in the heterospecific group (the second and third columns show the measures for each species, and the fourth column shows the measures between species) based on linear mixed model analysis.

Variable	Chinese bream	Qingbo	Chinese bream-Qingbo	Linear mixed model
Mean speed (cm s^−1^)	4.34 ± 0.67	4.34 ± 0.63		*F*_1,23_ = 0.278*P* = 0.603
Speed while moving (cm s^−1^)	4.99 ± 0.57	5.00 ± 0.59		*F*_1,23_ = 0.013*P* = 0.911
Synchronization of speed	0.84 ± 0.01	0.83 ± 0.01	0.84 ± 0.00	*F*_2,38_ = 0.970*P* = 0.388
Inter-individual distance (cm)	4.54 ± 0.21	5.06 ± 0.29	4.82 ± 0.23	*F*_2,38_ = 0.579*P* = 0.565
Percent time moving (%)	57.70 ± 3.42	57.42 ± 0.03		*F*_1,23_ = 0.025*P* = 0.876
Percent time swimming at the front (%)	19.07 ± 3.34	30.94 ± 3.34		*t*_1,14_ = − 2.516*P* = 0.025
Percent startle response (%)	93.75 ± 6.25	81.25 ± 9.15		*F*_1,7_ = 2.333*P* = 0.170
Startle response consistency (%)	87.50 ± 12.50	62.50 ± 18.30	81.25 ± 9.15	*F*_2,38_ = 1.371*P* = 0.266

**Notes.**

The mean swimming speed, speed while moving and percent time moving were measured individually with a total of 32 values for each variable. The linear mixed model included a fixed effect (species) and a random effect (group number) resulting in 1, 23 degrees of freedom. The percent startle response was measured at the whole group level for each species for a total of 16 values. The linear mixed model included a fixed effect (species) and a random effect (group number), resulting in 1, 7 degrees of freedom. The synchronization of speed, inter-individual distance and startle response consistency were variables describing and interaction between pairs of group members (i.e., between two Chinese bream, two qingbo and between Chinese bream and qingbo) for a total of 48 values for each variable, resulting in 2, 38 degrees of freedom. The percent time swimming at the front were measured at whole group level for a total of 16 values. However, the values did not meet the statistical assumptions for a linear mixed model. Therefore, the mean of each species was tested by an independent sample *t*-test, and the degrees of freedom were 1, 14.

### Polarization

There was no significant difference in polarization among the three treatment groups (Table 1).

### Distance between group members

The IID was significantly greater in the qingbo conspecific group than in the Chinese bream conspecific group and the heterospecific groups (Table 1) (ANOVA, *F*_2,20_ = 7.446, *P* = 0.004) (Fig. 1C). However, in the heterospecific group, there was no significant difference in IID among Chinese bream, qingbo and Chinese bream-qingbo (Table 2).

### Percent time swimming at the front

There was no significant difference in the CVs of the percent time swimming at the front of the group among the three treatment groups (Table 1). However, in the heterospecific group, the percent time swimming at the front of qingbo was significantly higher than that of Chinese bream (*P* = 0.044) (Table 2).

### Response to fright stimulus

There was no significant difference in the percent of startle response of fish among the three treatment groups (Table 1) and between Chinese bream and qingbo in the heterospecific group (Table 2). There was also no significant difference in the startle response consistency of fish among the three treatment groups (Table 1) and among Chinese bream, qingbo-Chinese bream and qingbo in the heterospecific group (Table 2).

## Discussion

### Shoaling coordination and cohesion in Chinese bream and qingbo

In the conspecific groups, qingbo showed higher mean swimming speed than Chinese bream. This was mainly due to the higher percent time moving of qingbo because the speed while moving did not differ significantly between the two conspecific groups. These results suggest that qingbo swam more often than Chinese bream, which may be partly due to the differences between the natural habitats of these two species: qingbo prefer to live in rapidly flowing water, whereas Chinese bream prefer to live in still water, although the two species may encounter each other occasionally in the field (Yan et al., 2013). Furthermore, qingbo and Chinese bream exhibited similar polarization and synchronization of swimming speeds. Previous study found the polarization of the black neon tetra (*Hyphessobrycon herbertaxelrodi*) higher than 85%, which was similar to that of Chinese bream and qingbo of the present study (about 90%, Table 1), while the polarization of the zebrafish (*Danio rerio*) was lower than 25% (Gimeno et al., 2016). The underlying mechanism of the profound difference between zebrafish (also a cyprinid species) and two species in the present study need further investigation. The early life history and test conditions such as water temperature, lighting conditions and group size also might all affect the polarization of a group.

Interestingly, Chinese bream spent more time swimming (similar to that of qingbo) in the heterospecific group. It is worth noting that the mean swimming speed changed parallel with percent time moving as swimming speeds while moving were similar between two species. Thus, both the mean swimming speed and percent time moving of Chinese bream increased to a level similar to that of qingbo in the heterospecific group. These results suggest that Chinese bream elevated their percent time moving, hence mean swimming speed to coordinate with qingbo in the heterospecific group, despite differences in body shape, behavior and physiology (Yan et al., 2013). Adjustment of swimming speed and percent time swimming is a precondition to the maintenance of a temporary mixed shoal in the field, which might benefit both species because they may share similar predators and food resources due to their similarities in trophic level and habitat. The observed change in mean swimming speed is consistent with a recent experiment that found that when group members with different spontaneous swimming speeds shoal together, Chinese bream individuals compromise and adjust their speed to a median speed (i.e., individuals with high speed lower their speed, whereas individuals with low speed elevate their speed), whereas among qingbo, only individuals with lower speed adjust their speed to match other individuals (Wu et al., 2017).

In addition to coordination, cohesion, as indicated by the IID between members, is an important group-level trait that has been examined in studies of fish schools (Herbert-Read et al., 2011; Katz et al., 2011). In the present study, the conspecific group of qingbo showed a greater distance between group members than the conspecific group of Chinese bream, suggesting an underlying difference between the two species’ shoaling cohesion. It was contrary to our expectation that more aggressive Chinese bream maintained shorter inter-individual distance from conspecifics than qingbo. One reason might be the lower percent time spent moving of Chinese bream compared to qingbo, as group members of both species show shorter inter-individual distance during resting compared to swimming status. Another explanation might be due to the context-dependent of aggressiveness; for example, the total amount of chasing of zebrafish during the feeding period increased strongly with the increase of feeding time under the limited food resources conditions (Grant & Kramer, 1992). In addition, the IIDs of all fish individuals were similar in the heterospecific group but shorter than those of qingbo in the conspecific group. The differences between Chinese bream and qingbo conspecific groups and between the Chinese bream conspecific and heterospecific groups are not due to the swimming activities (both mean swimming speed or percent time moving) as (1) Chinese bream in the heterospecific group spent more time moving and exhibited a higher mean swimming speed but similar IID compared to those in the conspecific group and (2) qingbo in the heterospecific group exhibited similar swimming activities but shorter IIDs compared to its conspecific group. Nevertheless, the present study demonstrated that in the heterospecific groups, the Chinese bream elevated their percent time moving, hence mean swimming speed while the qingbo decreased their IID to achieve consistent collective movement. As a result, the two species showed similar behavior in the mixed-species group. The underlying mechanism requires further investigation, and exploration of the interaction rules of collective motion among cyprinids with different morphological, physiological and ecological characteristics and the possible role of evolution might yield interesting results.

### Percent time swimming at the front among Chinese bream and qingbo

The CV of percent time swimming at the front did not differ between the two conspecific groups, in contrast to our expectations, as these species have differences in social behavior and preferred habitat. This observation is also in disagreement with the results of a study on golden shiners (*Notemigonus crysoleucas*), which showed that in some cases, more than 60% of time over the observed duration, the lead positions in group that consisted of 12 fish were consistently occupied by the same individuals (Reebs, 2000). Our results suggest that either there are only small differences in internal states, social hierarchies and/or social roles among group members in both species or that such differences are not reflected in position structure in the present study as four individuals were temporarily grouped and acclimated for only 5 min. However, qingbo preferred to swim at the front of the heterospecific group compared to Chinese bream, possibly due to the higher activity level of qingbo compared to Chinese bream. Once any individual (most likely qingbo) swam within the heterospecific group, other group members (most likely Chinese bream) would follow the initiator. The explanation may also interpret the difference in the percent time moving of Chinese bream in the heterospecific group compared with the conspecific group. However, researchers have argued that although there may be leaders in the sense that the same individuals are always found at the front (Mazeroll & Montgomery, 1995), the overall movement of the whole group is not necessarily determined by these individuals (Krause et al., 2000b). Nevertheless, the present study demonstrated differences in the positions occupied by group members in the heterospecific group. Whether such differences are consistent and, if so, the relationships between the position occupied and the internal state of a group member and/or the ecological benefit and cost of different positions warrant further investigation.

### The response to the artificial fright stimulus

Although there was no significant difference among the three treatment groups in the percent startle response, the percent startle response was approximately 30% and 40% lower in the Chinese bream conspecific group compared to the qingbo conspecific group and heterospecific group, respectively. It might be due to the higher activity level of qingbo and once any individual responds to a stimulus, other group members follow the initiator. However, the startle response consistency of the Chinese bream conspecific group was approximately 40% higher than those of the qingbo conspecific group and heterospecific group indicating that Chinese bream are more likely to reach a consensus decision than qingbo.

In conclusion, Chinese bream showed lower percent time moving and mean swimming speed than qingbo in conspecific groups, but this difference vanished as Chinese bream elevated their percent time moving, hence mean swimming speed to coordinate with a heterospecific group became similar to the traits of the qingbo conspecific group. These changes suggest that the two species share similar interaction rules regarding shoaling behavior. Qingbo maintained greater neighbor distances than Chinese bream in the conspecific group, but these differences vanished in the heterospecific group, and the IID became similar to that in the Chinese bream conspecific group, suggesting an underlying difference in the two species’ shoaling cohesion. The high synchronization of speed and polarization while moving are likely typical traits of collective movement. The CV of percent time moving at the front did not differ among the three treatments, but the percent time swimming at the front was higher among qingbo than Chinese bream in the heterospecific group, which might reflect the higher activity level of qingbo.

##  Supplemental Information

10.7717/peerj.3397/supp-1 Data S1 Raw data

## Additional Information and Declarations

### Data Availability

The following information was supplied regarding data availability: The raw data has been supplied as a Data S1.
